# Neonatal heel prick screening TSH concentration in the Netherlands as indicator of iodine status

**DOI:** 10.1186/s12937-021-00722-4

**Published:** 2021-07-04

**Authors:** Janneke Verkaik-Kloosterman

**Affiliations:** National Institute for Public Health and the Environment (RIVM), PO box 1, 3720 Bilthoven, BA The Netherlands

**Keywords:** Iodine status, New-born TSH, Heel prick screening

## Abstract

**Background:**

Neonatal Thyroid Stimulating Hormone (nTSH) is proposed as indicator of iodine deficiency in a population. Population’s iodine sufficiency is indicated by a proportion of the newborns less than 3% having nTSH above 5 mIU/L. The aim of this study was to explore the Dutch neonatal heel prick screening TSH data to assess iodine status in the Netherlands and identify determinants and potential confounders of this assessment.

**Methods:**

All newborns born in the Netherlands between 2007 and 2015 with a heel prick collection at day 3-7 were included (*n* = 1,435,600), except preterm neonates and baby’s with a low birth weight. Total T4 was measured for all children, nTSH was measured in the ~ 20% children with lowest total T4.

**Results:**

The proportion with nTSH > 5mIU/L fluctuated between 0.6-1.3% in 2007-2015. nTSH was significantly associated with laboratory performing the nTSH assay and age of heel prick sampling. The overall increasing trend in proportion nTSH >1mIU/L was confounded by the laboratories with different and changed assays.

**Conclusions:**

The low proportion neonates with high nTSH suggests a sufficient iodine status in the Netherlands. Whether the increased proportion nTSH>1mIU/L over the years is an early indicator of deterioration of the iodine status remains unclear, due to differences and changes in analytical assays. nTSH might be a valuable and inexpensive way to get crude insight in the (trend in) iodine status, but more research is needed on the validity and potential conditions.

**Supplementary Information:**

The online version contains supplementary material available at 10.1186/s12937-021-00722-4.

## Background

Adequate iodine intake is required for the production of thyroid hormones. These hormones regulate metabolic processes and play a crucial role in early growth and development of many organs, especially the brain [1]. Most growth and development of human brain happens during fetal life until 2-3 years postnatal life. Severe iodine deficiency in this period of life will result in irreversible mental retardation, and is also associated with other adverse effects including goiter, hypothyroidism, growth retardation, and increased risk of pregnancy loss [1, 2]. In addition, emerging evidence shows that also mild iodine deficiency in early life is associated with a (persistent) negative effect on the cognitive development of the child [1, 3–6].

In European countries, severe iodine deficiency is uncommon, but signs of mild-to-moderate iodine deficiency have been observed in several countries [7–11]. Although the iodine concentration in the soil is low in the Netherlands [12], in the 2007 the World Health Organization (WHO) global database on iodine deficiency, the Netherlands is one of the countries scored with an adequate iodine intake [13]. This is based on urinary iodine concentration of children (6-18 yrs.) measured sub-nationally in the Netherlands in 1995-1996 [14]. In the Netherlands there is no nation-wide monitoring program for iodine not for adults nor for children. A Dutch regional study showed that the urinary iodine excretion of adults decreased since 2008, however the iodine intake is still expected to be adequate [15, 16]. No recent iodine status data are available for Dutch children.

The Netherlands has a long history of iodization of salt, with bread being an important iodine source in the Dutch diet due to the addition of iodized salt [15]. Although mandatory fortification is legally not feasible in the Netherlands due to a court decision, mostly all bread is produced with iodized bakery salt containing 50-65 mg iodine per kg salt. This is due to a covenant [17] between the Ministry of Health and the conventional bakeries (industrial and traditional). For bakery products as well as bread replacing foods, a maximum concentration of 65 mg iodine per kg salt is allowed. Kitchen salt and foods other than bread may also voluntarily be produced with iodized salt, with a maximum concentration of 25 mg iodine per kg salt. However, for foods other than bread, iodized salt is used on a small-scale [15]. The observed decrease in urinary iodine excretion in Dutch adults in 2008, may be related to the effort to decrease the population’s salt intake. To reduce the population’s salt intake, the legal maximum amount of salt in bread was gradually reduced in the Netherlands, from 2.5% dry matter to 2.1% dry matter in July 2009 to 1.8% dry matter in January 2013. As a consequence, the iodine content of bread is decreased. However, no reduction in salt intake was observed in this period [16, 18]. In addition, an important factor is changes in the Dutch iodine policy to meet the European regulation on mutual recognition. The last revision of the Dutch iodine policy was in 2008 [19], resulting in an expansion of the foods allowed to contain iodized salt. Instead of a few food groups allowed to contain iodized salt (e.g. bread, processed meat products, kitchen salt), iodized salt may be added to all food groups, except unprocessed foods and drinks with > 1.2 vol% alcohol. The iodine levels in the specific food groups allowed to contain iodized salt prior to 2008, were set taking into account the iodine intake and consumption from these food groups. With the expansion of the foods that may contain iodized salt since 2008, the allowed maximum iodine content in salt was reduced with 7-38% compared to the situation prior to 2008. This was done to prevent too high iodine intakes if more foods potentially containing iodized salt would be consumed.

Measuring the urine iodine concentration is thought to assess the population’s iodine status adequately [20]. In addition, thyroid volume, serum thyroglobulin concentration and neonatal thyroid-stimulating hormone (nTSH) are proposed to assess a population’s iodine status [12, 21]. TSH seems only a sensitive indicator of iodine status in newborns. The thyroid of neonates has, in comparison with adults, a small iodine storage and a high iodine turnover rate. With a low iodine supply, increased TSH will maintain the high iodine turnover [22]. Sufficient iodine intake at the population level is indicated by less than 3% with a nTSH concentration exceeding 5 mIU/L. Higher proportions of 3-19.9% indicate mild iodine deficiency, of 20-39.9% indicate moderate deficiency, and above 39.9% indicate severe iodine deficiency [23]. The WHO proposed that existing newborn blood screenings programs can be used to assess iodine status in a population [1]. In addition, it can be used to study the effect of (changes in) iodination programs on the population’s iodine status.

The aim of this study was to examine the Dutch neonatal heel prick screening TSH data of 2007-2015 A) to estimate the percentage of blood spot TSH > 5 mIU/L to assess iodine sufficiency in the Netherlands in the years 2007 to 2015, and B) to pick-up a potential increase in nTSH values due to the known decrease in iodine intake in the Dutch adult population.

## Methods

### Data collection Dutch neonatal heel prick screening program

This study is a secondary data analyses of extant data. For this study we statistically re-analysed TSH and T_4_ data already collected and chemically analysed for the Dutch neonatal heel prick screening program for congenital hypothyroidism; no additional data was collected nor chemically analyses. A more detailed description of this screening program is provided by Verkerk et al. [24]. In brief, the Dutch nationwide neonatal screening program for congenital hypothyroidism (CH) was initiated in 1981. Neonatal heel prick blood was collected between 72 h to 168 h after the delivery. Blood samples were dried and analysed within 7 days at one of the five regional laboratories (i.e. Academic Medical Center, Amsterdam; National Institute for Public Health and the Environment, Bilthoven; IJsselland Hospital, Capelle a/d IJssel; St. Elisabeth Hospital, Tilburg; Isala Clinics, Zwolle).

The Dutch neonatal heel prick (blot spot) screening program is part of the National Population Screening Programme in the Netherlands funded by the State and falls under the Dutch Population Screening Act. For our study no additional ethical approval was necessary, because neither extra specimens nor additional chemical analyses were required, in addition the provided dataset was anonymised, and consent for additional scientific research was provided. The data was provided by the working group management information of Praeventis. Praeventis is the registration system of the Dutch national vaccine provision and prevention program, which includes the neonatal screening [25].

### Study population

Heel-prick samples of children born between January 1, 2007 and December 31, 2015 were included in the present study. This resulted in a total sample of 1,611,391 children, which varied between 171,138 children in 2015 to 185,271 children in 2009. Participation rate was high; > 99% [26, 27].

Children with missing data for T_4_ or nTSH concentration, age at heel prick sampling, pregnancy duration, birth weight or laboratory were excluded (*n* = 47,380; 2.9%). In addition, children with a heel prick at < 3 days or > 7 days of age were excluded (*n* = 17,618; 1.1%), as well as children with a low birthweight (≤2500 g, *n* = 50,165; 3.1%) and children born preterm (gestational age ≤ 36 weeks, *n* = 59,679; 3.7%), since these factors may influence nTSH concentration [28]. Children with a coded birth weight (7.77 kg or more) and pregnancy duration (> 45 weeks) were excluded, as these numbers are not reflecting actual birth weight and pregnancy duration (*n* = 60,628; 3.8%). The final study sample after exclusions consisted of 1,435,600 children (i.e. 89% of the total sample).

### Analysis of dried blood samples

In the Netherlands, the screening for CH is different compared to many other countries [29]. In most countries newborns are screened for primary CH (defect in thyroid) only. In these countries, nTSH is measured first, subsequently total T_4_ or T_4_ is measured only in infants with raised nTSH. In the Netherlands, besides primary CH newborns are also screened for central CH (defect in hypophysis or hypothalamus) and therefore, total T_4_ is measured first, followed by nTSH in the infants with lowest total T_4_ [24]. In the screening for CH, total T_4_ (thyroxin) concentration, expressed as the number of standard deviations (SD) from the daily average, was determined for all infants (further referred to as ‘total population’). For samples with a total T_4_ concentration ≤ − 0.8 SD, i.e. circa 20% of all newborns, nTSH concentration (mIU/l whole blood) was also determined from the same filter paper cards with dried blood spots (further referred to as ‘TSH-subpopulation’) [24]. In addition, for newborns with a total T_4_ concentration ≤ − 1.6 SD (about 5% of all infants) also thyroid-binding globulin (TBG) was determined. According to the screening procedures, TSH estimates were available as integers. Children with very low T_4_ concentration or very high nTSH concentration were referred to a paediatrician. A second heel prick was performed if there were slight deviating T4:TBG ratio or nTSH concentrations [24].

Total T_4_ and nTSH concentrations were analysed in 3.2 mm punches of blood spots on newborn screening filter paper cards. As of January 1, 2007, concentrations of T_4_ were determined with an automated assay using the AutoDelfia or GSP platform (PerkinElmer, Turku, Finland), and concentrations of nTSH with these same platforms (laboratory Zwolle: AutoDelfia; laboaratories Bilthoven, Capelle a/d IJssel, Tilburg AutoDelfia later GSP platform) or a commercial manual ELISA (laboratory Amsterdam; Thermofisher Scientific, Waltham, MA). The analytical methods used in the screening program have gradually been replaced as they became obsolete and cut-off values have been updated. Since 2010 for T_4_ the SD is not calculated based on the daily average, but is based on a standard value for the variation coefficient in the population of 22%. In February 2012 the Bilthoven laboratory, and in April 2013 the laboratories in Capelle a/d IJssel and Tilburg exchanged the AutoDelfia for the GSP platform. As results from the GSP platform were lower than those measured with the AutoDelfia, for harmonisation of the results TSH concentrations were multiplied with a factor of 1.25. After re-evaluation the use of the nTSH factor lapsed since May 2016. Therefore, in our study we used the uncorrected nTSH-values.

If the T_4_ or nTSH measurement was not reliable, for example due to insufficient filling of the array, a second measurement was collected. Since the second measurement was considered more accurate, this estimate was included in our analyses.

### Statistical analyses

Blood spot total T_4_ and nTSH were tabulated by year of birth, gender, age at heel prick sampling, birth weight, pregnancy duration, season at heel prick sampling, and laboratory. For T_4_ this was done separately for the total population and the nTSH-subpopulation.

Analyses involving group comparison were performed using Kruskall-Wallis H test, those involving two continuous variables were performed using Spearman correlation. Associations were illustrated using histograms. Statistical analyses for which the magnitude of the differences was small (Kruskal-Wallis H test: χ^2^ < 150 or Spearman correlations: rho < 0.05) were considered not relevant, even though these might be statistically significant based on the *p*-value. Cochrane Mantel-Haenzel test was performed to study if there was a trend over the calendar years between a category of TSH and each category of the characteristics found to be statistically significant in the previous described analyses. All statistical analyses were performed at a 5% level of significance, which was Bonferonni-adjusted for multiple testing to 0.19% (i.e. 0.05 divided by 27 different analyses). All statistical analyses were performed with SAS software version 9.4 (SAS Institute Inc., Cary, NC).

Univariate logistic regression was applied to estimate the effects of year of birth, gender, age at heel prick sampling, birth weight, pregnancy duration, season at heel prick sampling, laboratory on the change of TSH exceeding a pre-specified cutoff point. Results were only presented for the characteristics considered relevant (see above). A cutoff of 5 mIU/L was chosen, as this is the cut point provided by WHO to assess iodine status using nTSH. In addition, a cutoff of 1 mIU/L was selected, as the raw data indicated a potential change over the years in the categories with 1 and 2 mIU/L. To correct for potential confounders, also multivariate logistic regression was applied, taking all earlier studied characteristics into account, as well as relevant interaction terms.

## Results

### General characteristics of the population

Of the children included in this study, 51% were male, almost 90% of heel prick blood was sampled at the age of 4-6 days, median birth weight was 3.5 kg, and median gestational age was 280 days (i.e. 40 weeks; Table 1). Overall most births were in autumn and there was some fluctuation over the years (Table 1 and Online Supplemental Material Table 1). For birth season, age heel prick, and laboratory there was a statistically significant fluctuation over the years (Online Supplemental Material Table 1). However the differences over the years were small with an exception of year 2010 in which there were more children born in winter and less in summer compared to the other years. Over the years, the proportion of heel prick sample collected on day 3 increased from 4 to 10%. This increase of almost 55% was accompanied by a decrease of 4% in the proportion of heel prick sample collection on day 4, 5, and 6 and a decrease of 60% on day 7 (Online Supplemental Material Table 1). Overall the laboratories of Tilburg and Zwolle analysed the lowest proportion of measurements (Table 1). These proportions were similar for most years, with the exception of 2009 and 2010 with fewer samples being analysed in the laboratory of Tilburg. (Online Supplemental Material Table 1).

**Table 1:** Characteristics of the study population presented as count (sample size), median (5th-95th percentile: pregnancy duration, birth weight), or percentage (gender, season at birth, age at heel prick, laboratory)

N		total population		nTSH subpopulation	
		1,435,600		264,407	
**Gender (%)**	**male**	51	χ^2^ = 1017, 1 d.f., *p* < 0.0001	59	χ^2^ = 9074, 1 d.f., *p* < 0.0001
**Season at birth (%)**	**spring**	24	χ^2^ = 2850, 3 d.f., *p* < 0.0001	24	χ^2^ = 826, 3 d.f., *p* < 0.0001
	**summer**	24		24	
	**autumn**	27		27	
	**winter**	25		25	
**Age at heel prick (%)**	**day 3**	8	χ^2^ = 1,007,684, 4 d.f., *p* < 0.0001	7	χ^2^ = 148,329, 4 d.f., *p* < 0.0001
	**day 4**	50		46	
	**day 5**	24		24	
	**day 6**	15		19	
	**day 7**	3		4	
**Laboratory (%)**	**Amsterdam**	20	χ^2^ = 34,673, 4 d.f., *p* < 0.0001	20	χ^2^ = 6307, 4 d.f., *p* < 0.0001
	**Bilthoven**	21		21	
	**Capelle**	25		25	
	**Tilburg**	17		17	
	**Zwolle**	17		17	
**Pregnancy duration (days)**		280 (262-292)		277 (260-291)	
**Birth weight (kg)**		3.5 (2.8-4.3)		3.4 (2.7-4.2)	

The nTSH-subpopulation was about 20% of the total study population, as expected. In the nTSH-subpopulation, the proportion male was higher (59%), as well as the proportion with collection of heel prick sample on day 6 and 7 (Table 1) in comparison with the total population. In addition, the pregnancy duration was shorter (median 277 days) compared to the total population. The fluctuations over the years for the studied characteristics in the nTSH-subpopulations were similar to the fluctuations over the years for total population (Online Supplemental Material Table 1).

### Neonatal T_4_ levels

The median (P5-P95) total T_4_ showed some variation over the calendar years and varied from 83 (57-118) nmol/l in 2008 to 91 (62-128) nmol/l in 2015 (Table 2). This value was lower for male than female (*p* <  0.0001 each year, Online Supplemental Material Table 2). In addition, the median total T_4_ level increased with birth weight (*p* <  0.0001 each year) and pregnancy duration (*p* <  0.0001 each year) and was lower for data collected on day 6 or 7 compared to days 3-5 (*p* <  0.0001 each year). There was no relevant variation over the years for total T4 and each of the studied characteristics (Online Supplemental Material Table 2).

**Table 2:** Dried blood spot total T_4_ concentration (nmol/L) per calendar year (median (P5-P95) for total neonatal population and TSH subpopulation (Kruskall-Wallis)

	Total T_**4**_ (nmol/L)									***p***-value
	2007	2008	2009	2010	2011	2012	2013	2014	2015	
**Total population**	87 (58-126)	83 (57-118)	85 (58-120)	90 (63-127)	88 (61-125)	87 (60-123)	86 (60-122)	86 (59-122)	91 (62-128)	χ^2^ = 40, 8 d.f., *p* < 0.0001
**TSH-subpopulation**	64 (48-77)	62 (46-74)	64 (47-77)	68 (52-79)	66 (50-78)	65 (49-77)	65 (49-77)	65 (48-76)	68 (51-80)	χ^2^ = 150, 8 d.f., *p* < 0.0001

As expected, the median (P5-P95) total T_4_ was lower for the nTSH-subpopulation compared to the total population of new-borns, as they were selected based on lowest total T_4_. The median total T_4_ showed some variation over the calendar years ranging from 62 (46-74) nmol/l in 2008 to 68 (52-79) nmol/l in 2010 (Table 2), however based on the small χ^2^ this was not considered relevant. In this nTSH-subpopulation, the difference in total T4 was not as pronounced as in the total population for gender, birth weight, and pregnancy duration. (Online Supplemental Material Table 2). Similar to the total population, there was no relevant variation over the years for total T4 and each of the studied characteristics in the nTSH-subpopulation (Online Supplemental Material Table 2).

### Distribution of nTSH-levels

**Fig. 1:**
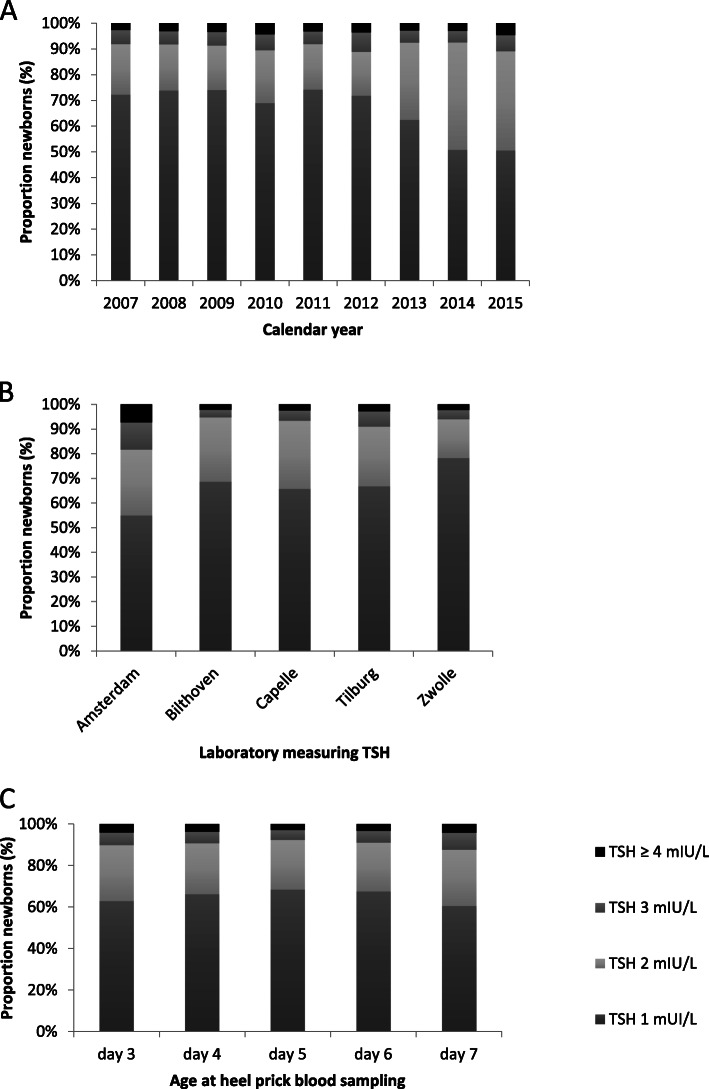
Distribution of rounded nTSH values measured in heel prick blood of new-borns in the Netherlands, **A** in each calendar year – Kruskal-Wallis H test χ^2^ = 8149 *P* < 0.0001, **B** for each laboratory – Kruskal-Wallis H test χ^2^ = 7195 *P* < 0.0001, **C** across ages at heel prick sampling – Kruskal-Wallis test χ^2^ = 482 *P* < 0.0001

In each calendar year, about 90% of the nTSH-subpopulation had a rounded nTSH value of 1 or 2 mIU/L. The Kruskal-Wallis H test showed that there was a statistically significant difference in nTSH levels between the calendar years (χ^2^ = 8149, 8 d.f., *p* <  0.0001). Most children had a rounded nTSH value of 1 mIU/l, however this percentage decreased from 69 to 74% in the calendar years 2007-2012 to 50% in 2015. With this decrease there was a joint increase in children with a rounded nTHS level of 2 mIU/L; 17-21% in 2007-2012 to 39-42% in 2014-2015. The percentage children in each of the higher nTSH categories (3mIU/L or higher) remained below 8% (Fig. 1a & Online Supplemental Material Table 3a). In addition there was a statistically significant difference in nTSH levels between the laboratories (Kruskal-Wallis H test: χ^2^ = 7195, 4 d.f., *p* <  0.0001). The nTSH distributions were similar for the laboratories in Bilthoven, Capelle and Tilburg, but the nTSH values in Zwolle tended to be lower (especially in 2012-2015) and those in Amsterdam higher (Fig. 1b & Online Supplemental Material Table 3b). The distribution of nTSH levels differed significantly across the ages at heel prick sampling (Fig. 1c & Online Supplemental Material Table 3c; Kruskal-Wallis H test: χ^2^ = 482, 4 d.f., *p* <  0.0001), with the lowest nTSH values at day 5. For the characteristics gender, pregnancy duration, birth weight, and season statistical significant differences (*P* <  0.0001) in nTSH values were observed, however the magnitude of the differences was small (Kruskal-Wallis H test: χ^2^ < 150 or Spearman correlations: rho < 0.05) and therefore considered not relevant. The proportion new-borns with nTSH value equal to 1 mIU/L over the calendar years varied statistically significantly across laboratories (Cochran Mantel-Haenzel test χ^2^ = *p* <  0.0001 (Table 3). The laboratories Bilthoven, Capelle, and Tilburg showed a steep decrease in the proportion new-borns with nTSH = 1 mIU/L, however in the laboratory of Zwolle there was a small decrease since 2010 which remained constant in later years. Over the years the proportion new-borns with nTSH = 1mIU/L measured in the laboratory of Amsterdam showed first an increase and in the last two years a decrease. The proportion new-borns with a nTSH value equal to 1 mIU/L over the calendar years varied statistically significantly across ages of heel prick sampling (Cochran Mantel-Haenzel test χ^2^ = *p* <  0.0001). A decrease in percentage of neonates with a nTSH value of 1 mIU/L was observed for day 3-6 (Table 3). For day 7, this percentage showed first an increase and then a decrease.

**Table 3:** Proportion (%) new-borns with TSH = 1 mIU/L in heel prick blood by laboratory and calendar year and by age at heel prick screening and calendar year (both Cochran-Mantel-Haenzel test)

	2007	2008	2009	2010	2011	2012	2013	2014	2015	***p***-value
**Laboratory**										
Zwolle	78.74	85.66	84.54	74.08	76.38	75.45	77.37	75.17	75.47	**< 0.0001**
Tilburg	83.84	81.77	74.96	78.16	78.52	69.46	55.27	39.9	44.21	**< 0.0001**
Capelle	76.16	78.85	77.18	73.29	72.56	69.99	57.71	40.6	48.98	**< 0.0001**
Bilthoven	80.02	84.64	80.02	73.87	78.36	69.11	52.84	43.25	50.72	**< 0.0001**
Amsterdam	43.2	39.04	55.11	48.78	65.2	75.85	72.76	62.55	36.25	**< 0.0001**
**Age**										
day 3	71.27	74.62	74.77	66.58	69.64	67.69	59.02	47.00	49.81	**< 0.0001**
day 4	72.71	75.73	74.76	69.55	73.08	69.79	60.21	48.97	50.30	**< 0.0001**
day 5	74.19	76.27	76.37	71.59	76.04	73.57	64.05	51.89	51.92	**< 0.0001**
day 6	72.08	72.00	70.72	66.78	75.46	75.66	67.33	55.27	50.41	**< 0.0001**
day 7	61.44	52.78	65.08	59.22	79.39	73.92	65.44	51.93	44.94	**0.0004**

**Fig. 2:**
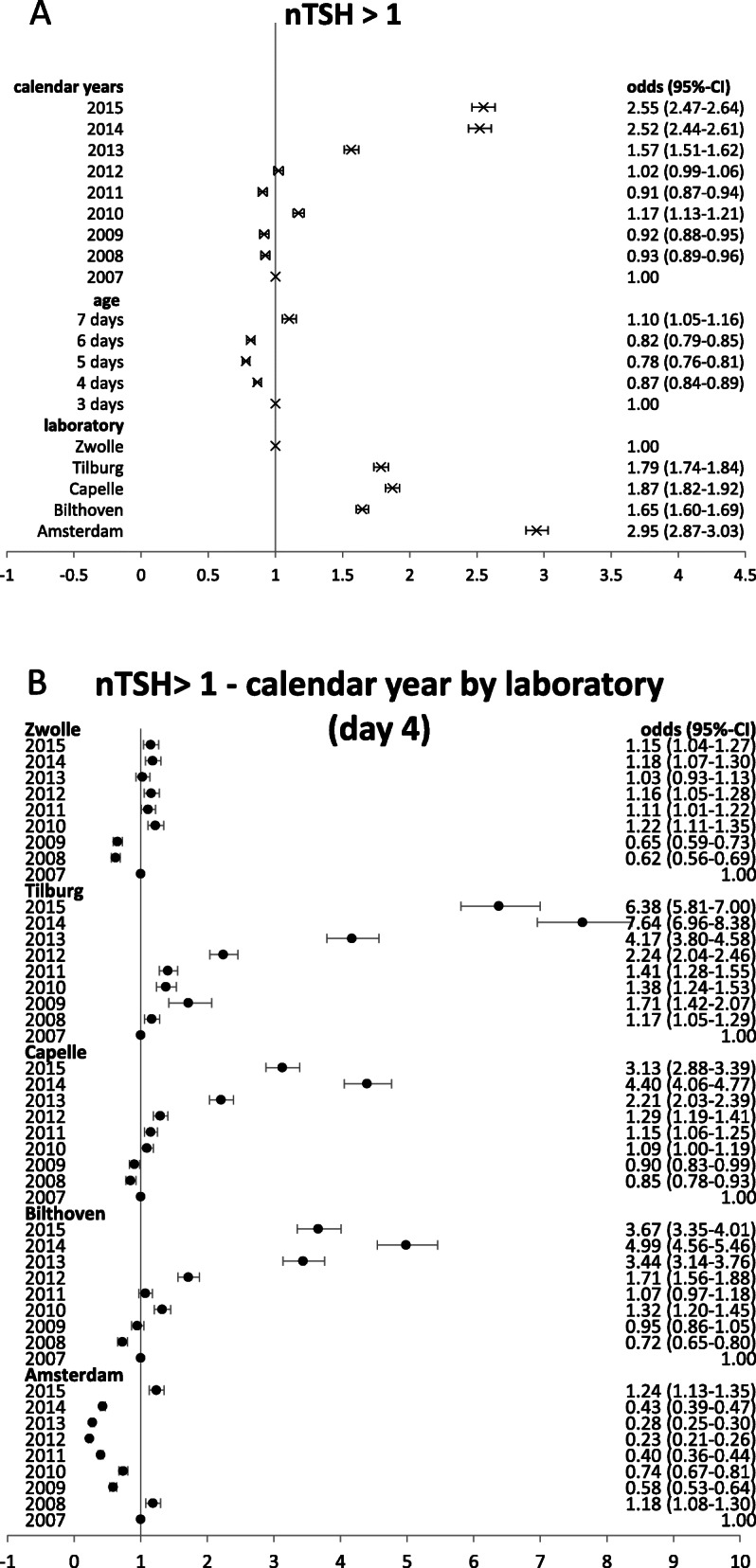
Odds ratio and their 95% confidence intervals of exceeding blood spot screening cut off (nTSH >1mIU/L versus nTSH = 1mIU/L) for several characteristics, **a** univariate analyses, **b** multivariate analyses with the following characteristics (reference value): year (2007), laboratory (Zwolle), age of heel prick blood sampling (4 days), gender (male), birth weight category (middle: 3.31-3.71 kg), pregnancy duration category (middle: 276-284 days), season (spring), and interaction terms: year*laboratory, age*year, age*laboratory; presented for age = day 4

The observed changes in the distribution of proportions with a specific rounded nTSH value for the different categories of calendar year, laboratory, and age at heel prick sampling were quantified in univariate logistic regression models. As in the Figs. 1a-c the largest differences were observed for the category nTSH 1 mIU/L, logistic regression was applied for nTSH > 1mIU/l versus nTSH = 1 mIU/L. Until 2012, there were relatively small differences in percentage with nTSH > 1 mIU/L (odds ratio 0.9-1.2). Starting at 2013, there was an increase in percentage of children with nTSH > 1 mIU/L (odds ratio 1.6-2.6), which seemed to stabilize in 2014-2015 (Fig. 2). The percentage nTSH > 1 mIU/L was highest for Amsterdam (odds ratio 2.95 compared to Zwolle), also the laboratories Bilthoven, Capelle, and Tilburg had a higher percentage compared to Zwolle (odds ratio 1.65-1.87). The percentage children with nTSH > 1 mIU/L was lowest for day 5 (odds ratio 0.78 compared to day 3), and highest for day 7 (odds ratio 1.1 compared to day 3) (Fig. 2a). Results from a multivariate logistic regression model showed that there was a statistically significant interaction between year and laboratory (*p* < 0.0001), laboratory and age (*p* < 0.0001), and year and age (*p* = 0.0004). Figure 2b shows the odds ratios for the probability that nTSH > 1 mIU/L of the calendar years per laboratory for age of heel prick sampling is 4 days. Although there were differences in odds ratio values for the trend over the years for each laboratory per age of heel prick blood sampling, the pattern over the years was similar for each age of heel prick blood sampling for a specific laboratory (data not shown). For the laboratories of Bilthoven, Tilburg, and Capelle the trend over the years was similar, with an increasing odds ratio starting at 2009-2010 (Fig. 2b). This means that from 2009 to 2010 the proportion children with nTSH > 1 mIU/L increased compared to 2007. For the laboratory of Zwolle also an increase in the proportion children with nTSH > 1 mIU/L was observed starting at 2010, however it remained more or less stable in the period 2010-2015 (odds ratio range 1.03-1.22). In contrast, the laboratory of Amsterdam first showed a decrease in the proportion children with nTSH > 1 mIU/L compared to 2007, and starting round 2013 this proportion seemed to increase compared to the years before. In general the proportions with nTSH> 1 mIU/L were higher for Amsterdam compared to the other laboratories (Table 3).

### Evaluation of the population’s iodine status

According to WHO, iodine intake at the population level is sufficient if the percentage of nTSH concentration exceeding 5 mIU/L is below 3% [23]. The proportion with a nTSH value > 5 mIU/L almost doubled in our sample, from about 0.6% in 2007 to 1.1% in 2015 (Online Supplemental Material Table 3a). However, there is variation over the calendar years, with a marked high percentage of 1.3% in 2010. In addition, for age at heel prick sampling and laboratory there were variations in the proportion with a TSH-value > 5 mIU/L (Online Supplemental Material Tables 3b-c). Over the calendar years there was a trend in increasing proportion of new-borns with nTSH > 5mIU/L in the laboratories of Tilburg (Cochran Mantal-Haenzel value 9.7894, *p* = 0.0018), Capelle (Cochran Mantal-Haenzel value 4.0181, *p* = 0.0450), and Bilthoven (Cochran Mantal-Haenzel value 5.8333, *p* = 0.0157) (Table 4). For the other laboratories, no such trend was observed. In each calendar year, the proportion with nTSH> 5 mIU/L remained below 3%. However, in the laboratory of Amsterdam this proportion was around 2.5% in 2010 and 2015, in the other calendar years it was between 0.96 and 1.59%. There was no statistically significant trend over the calendar years between nTSH > 5 mIU/L and age at heel prick sampling (Table 4), except for day 7 (Cochran Mantal-Haenzel value 4.7671, *p* = 0.0290).

**Table 4:** Proportion (%) new-borns with nTSH >5 mIU/L in heel prick blood by laboratory and calendar year and by age at heel prick sampling and calendar year (both Cochran-Mantel-Haenzel test)

	2007	2008	2009	2010	2011	2012	2013	2014	2015	***p***-value
**Laboratory**										
Zwolle	0.45	0.67	0.50	0.93	0.73	0.72	0.48	0.62	0.55	0.8723
Tilburg	0.38	0.55	0.71	0.87	0.69	0.96	0.93	0.69	0.78	**0.0018**
Capelle	0.44	0.63	0.72	0.97	0.80	0.96	0.77	0.69	0.78	**0.0450**
Bilthoven	0.46	0.50	0.81	0.96	0.78	0.69	0.81	0.91	0.76	**0.0157**
Amsterdam	1.40	1.59	1.55	2.44	1.41	1.31	0.96	1.14	2.58	0.3723
**Age**										
day 3	0.81	1.13	0.97	1.7	0.62	0.95	0.89	0.99	1.28	0.8156
day 4	0.8	0.9	1.01	1.35	1.09	1.01	1.02	0.91	1.16	0.0681
day 5	0.54	0.7	0.71	0.85	0.67	0.91	0.5	0.78	0.93	0.0529
day 6	0.47	0.55	0.92	1.41	0.65	0.73	0.58	0.5	0.94	0.4966
day 7	0.15	0.76	0.64	1.33	1.02	0.89	0.36	0.77	1.1	**0.029**

**Fig. 3:**
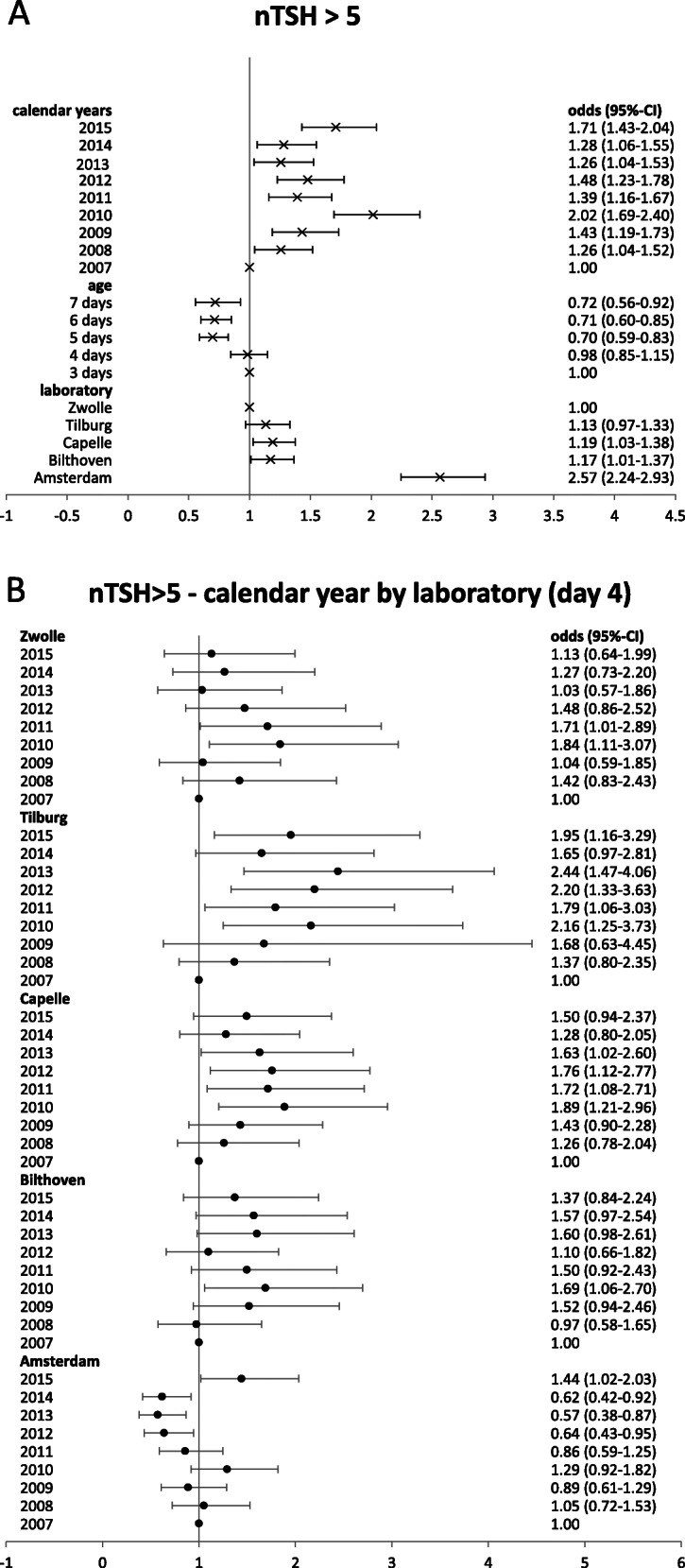
Odds ratio and their 95% confidence intervals of exceeding blood spot screening cut off (nTSH >5mIU/L versus nTSH<=5mIU/L) for several characteristics, **a** univariate analyses, **b** multivariate analyses with the following characteristics (reference value): year (2007), laboratory (Zwolle), age of heel prick blood sampling (4 days), gender (male), birth weight category (middle: 3.31-3.71 kg), pregnancy duration category (middle: 276-284 days), season (spring), and interaction terms: year*laboratory, age*year, age*laboratory; presented for age = day 4

These changes in nTSH> 5mIU/L were also quantified with univariate logistic regression, showing a higher percentage with nTSH > 5 mIU/L at day 3 and 4 (odds ratio 0.98-1.00) compared to days 5-7 (odds ratio 0.70-0.72; Fig. 3a). The percentage with nTSH > 5 mIU/L was in the laboratory of Amsterdam higher compared to the other laboratories; odds ratio 2.57 compared to the laboratory in Zwolle (Fig. 3a). Results from a multivariate logistic regression model showed that there was a statistically significant interaction between year and laboratory (*p* < 0.0001). Figure 3b shows the odds ratios for the probability that nTSH > 5 mIU/L for each of the calendar years per laboratory for age of heel prick sampling is 4 days. Although there were differences in odds ratio values for the trend over the years for each laboratory per age of heel prick blood sampling, the pattern over the years was similar for each age of heel prick blood sampling for a specific laboratory (data not shown). Except for day is 7 for which the odds ratio values were in general higher compared to the other ages. In addition, for each lab in most years the odds ratio was statistically significantly above 1 (Online Supplemental Material Figure 1). In all laboratories, except Amsterdam, the odds ratios showed an increase starting in 2008-2009 compared to the reference year 2007. However this was not statistically significant in each year and for each laboratory (Fig. 3b). This means that the proportion children with nTSH > 5 mIU/L tended to be (non-statistically significant) higher in those years compared to 2007. In the Amsterdam laboratory the proportion children with nTSH. 5 mIU/L was higher compared to 2007 in 2010 and 2015 (statistically significant).

## Discussion

Based on nTSH as indicator to assess a population’s iodine status [23], the Dutch population is considered iodine sufficient between 2007 and 2015, with a prevalence of nTSH > 5 mIU/L below 3%, namely ranging at 0.6-1.3% depending on calendar year. These results confirm studies on urinary iodine content performed in a similar period in Doetinchem, the Netherlands [18]. In 2006, 2010 and 2015 the median 24-h iodine excretion was > 150 μg/d indicating an adequate iodine intake in the Dutch adult population. In several other countries nTSH was used to assess the population’s iodine status as well. Our results are similar to Northern Ireland with an overall proportion of 0.49% with nTSH > 5 mIU/L in 2003-2014 [30]. But higher proportions were observed in Belgium 2.6-3.3% in 2009-2011 [9], Australia 4.1-9.7% in 2001-2006 [31], Germany 14% in 2005-2006 [32], and Latvia 8.4-16.5% in 2000-2002 [33]. Variation in iodine intake, but also differences in assessment of nTSH (e.g. sampling time, analytical method) or the cohort composition could be explanations for the observed variation. It is earlier indicated that several factors are associated with nTSH levels [28, 34, 35]. In our study, nTSH was statistically significantly associated with the laboratory performing the nTSH assay, age of heel prick sampling, gender, pregnancy duration, birth weight, and birth season. The magnitude of the differences was small for gender, pregnancy duration, birth weight and birth season, indicating that these were not the most relevant influencing factors in our data, but probably identified due to the large sample size.

For interpretation of the results and comparability between studies it is important to address some factors that potentially could have influenced the results. To minimize the effect of interfering factors in our study, we excluded premature born babies, as well as very early and late collection of heel prick blood samples. WHO states that heel prick blood samples should be taken on day 3 or 4 [1, 36]. Similar to our study, several others used a more brought range of sampling days [9, 11, 37]. If we limit our analyses to day 3 and 4, the proportions with nTSH > 5 mIU/L increased a little from 0.6-1.3% to 0.8-1.4%, but remained overall below 3%. Only for the laboratory of Amsterdam in 2015 the proportion increased to 3.1%, which may indicate a potential mild iodine deficiency.

Based on conflicting conclusions regarding the iodine status studied with nTSH or urinary iodine concentration, some authors state that the current cut-off of 3% frequency of nTSH > 5mIU/L is not sensitive enough to detect mild iodine deficiency [9, 38].

In some studies a lower cut off of 2 mIU/L is proposed to detect mild iodine deficiency. In our study the proportion children with nTSH > 2 mIU/L ranged in 2007-2015 from 7.6-11.3%. The proportions found in cohorts in Northern Ireland (6.2%) [30] and Wales UK (10.9-11.9%) [37] are similar. Higher proportions were observed in Belgium (21-40%) [9]. It remains unclear how to interpret these results, as the Netherlands is considered iodine sufficient and for the other areas mild iodine deficiency among pregnant women is reported based on urinary iodine concentration. Also other issues may have resulted in the mentioned discrepancy of results between studies based on nTSH and urinary iodine concentration, for instance issues interfering with nTSH levels, besides maternal iodine intake, as described above. On the other hand, even though urinary iodine concentration is considered as a standard to assess population’s iodine status there are also factors influencing the results. For instance using WHO’s cut-offs for iodine concentration it is assumed that the mean urinary volume is 1.5 L for adults an 1 L for children; this is not the case for all populations [16, 39]. Higher urine volumes will dilute the iodine concentration what may result in incorrect conclusions on inadequacy. More research is required to get insight in the best way to interpret the nTSH values for predicting the population’s iodine status. As far as we know no validity study is available providing a more absolute numeric value for the interpretation of nTSH in relation to population’s iodine status, as indicated earlier by Burns et al. [40].

The second aim of our study was to pick up the reduction in iodine intake in the Dutch adult population in nTSH levels. As changes in the nTSH distribution may provide early indications of deterioration of the iodine status in pregnant women [9, 37, 40, 41], and could be interpreted as an early warning and a need for regular monitoring. Due to changes in the Dutch iodine policy in 2008 and 2012, as mentioned in the introduction, the maximum iodine level of iodized salt was reduced in order to allow more food groups to be produced with iodized salt without increasing the risk of excessive intakes [19]. The Dutch iodine intake of adults estimated from 24-h urine collections decreased between 2006 and 2010 on average 30% and remained stable until 2015 [18]. In Switzerland a 25% increase in iodine content of table salt improved the iodine status, which was observed in a decrease in proportion nTSH > 5 mIU/L of 2.9 to 1.7% [36]. In our study, an increase in proportion with nTSH> 1mIU/L was observed for four of the five laboratories, which might indicate a deterioration of the iodine status. For the proportion with nTSH>5mIU/L no clear significant trend could be found. However, the variable laboratory was an important confounding factor and there was a strong interaction between laboratory and year. In our study, the analyses were therefore also showed results for each laboratory separately and used a multivariate regression model. The analytical measurements were performed with three different methods. Li et al. (2010) showed that there is a large range of nTSH assays with a broad variation in assay performance [34]. This will make it difficult to compare results measured with different assays. For three laboratories (Bilthoven, Capelle a/d IJssel, Tilburg) the analytical assay did change during the studied time-period and therefore a potential change in nTSH distribution can be related to change in iodine intake, but also to the new assay. In addition, as described in the methodology section, in the first years of introduction of the new assay, the nTSH levels were lower compared to the prior assay. However, this difference disappeared in later years and the earlier introduced correction factor was lapsed. This might be related to bias due to assay performance within the same assay, which remains unclear [34]. On the other hand, the increase in the proportion with nTSH > 1 mIU/L for these three laboratories started around 2009, prior to this analytical change. For two laboratories the analytical assay was equal over the studied years, but different between the laboratories. A statistical significant increase in the proportion with nTSH>1mIU/L was found for the Zwolle laboratory starting round the year decreased iodine intake in the Dutch population was expected. This might indicate deterioration of iodine intake. However, no such trend was observed for the Amsterdam laboratory. It therefor remains unclear if the decreased iodine intake in the Dutch population would have affected the nTSH levels and could be picked-up.

The laboratory in Amsterdam, had the highest yearly proportion new-borns with nTSH > 5 mIU/L of about 1.0-2.6%. In the other four laboratories this proportion was lower, varying from 0.4-1.0%. It remains unclear whether this difference in proportion nTSH > 5 is solely due to the different laboratory assays or that the population new-borns measured in the laboratory of Amsterdam do differ from the rest of the country. Based on the characteristics available in our study no differences between the Amsterdam region and the rest of the country could be identified explaining the larger proportion. In the most recent Dutch National Food Consumption Survey (2012-2016) no large differences in food consumption were observed between regions in the Netherlands [42].

Besides the already mentioned different nTSH assays in the laboratories and the change in assay in some laboratories this study has other limitations. The nTSH assays are developed for CH screening in new-borns. The cut-off values applied in this screening are higher (e.g. 10-20 mIU/L) than the cut-off value for assessing iodine status (> 5 mIU/L). The performance of the assays may be different for these values. In addition, in the Dutch screening for congenital hypothyroidism, total T_4_ is measured for all neonates, and nTSH only for the circa 20% with the lowest total T_4_ concentrations. As total T_4_ concentration is associated with several characteristics in the study population, the nTSH-subpopulation is no representative sample of the total population (selection bias, see also Table 1). As total T_4_ concentration and nTSH are associated, it is expected that the proportion children with an elevated nTSH is to some extend overrepresented in this group. However, there are no indications that the overall associations with calendar year, day of heel prick sampling, and laboratory would different for both groups. Another limitation is the rounded nTSH values. For the congenital hypothyroidism screening, there was historically no need to retain the digits. To assess iodine status via nTSH it is recommended to retain some digits, to be able to better predict the proportions with a nTSH concentration above a specified cut-off level. For example, in our study, the proportion with TSH > 5 mIU/L is an underestimation, as it does not include children with a nTSH concentration between 5.01 and 5.5 mIU/L, as these were rounded to 5 mIU/L. Besides limitations this study has also some strengths. One strength is that a very large part of the newborns is included in the Dutch heel prick screening (> 99%), and that we have data available from a large time period (2007-2015). This made it possible to study the trend over time as well as performing sub-group analyses. Earlier Burns et al. stated that a trend in nTSH over years is informative in addition to the yearly numeric values [40]. Based on the results from our study we support this, given that the same nTSH assay is used over the years.

Based on the discussed limitations of the nTSH-marker, it is recommended to not base a conclusion on iodine status on this biomarker solely, but in combination with other biomarkers for population iodine status, e.g. repeated 24-h urinary iodine excretion of pregnant women in the Netherlands. Although urinary iodine concentration or 24-h urinary iodine excretion are generally proposed as most valid measurements, the assessment with these measures also have some assumptions or difficulties in the interpretations. For example the earlier mentioned assumption of a urinary volume of 1.5 L/d. As the nTSH-data from the Netherlands is readily available from the congenital hypothyroidism screening it can be used as a crude indicator for the iodine status in the Netherlands. To make it better usable for studying iodine status, it is recommended to retain the unrounded values for total T4 as well as nTSH. As pregnant women are vulnerable for iodine deficiency, due to increased requirements and the irreversible effects on the child’s cognition, it is important to monitor their iodine status regularly.

## Conclusion

The results show that the iodine status seems sufficient in the Netherlands. Whether the increased proportion nTSH>1mIU/L could be interpreted as early indications of a deterioration of the iodine status remains unclear, mainly due to differences and changes in laboratory assays. Unless the flaws, next to other markers for iodine status, nTSH measured for congenital hypothyroidism screening might be valuable and inexpensive data to get a crude impression of the iodine status and the trend in time. But more research is needed to validate nTSH as indicator of population’s iodine status and the potential conditions.

## Supplementary Information

**Additional file 1.**

## Data Availability

The data analyzed during the current study are available from Praeventis, but restrictions apply to the availability of these data, which were used under license for the current study, and so are not publicly available.

## Abbreviations

nTSH: neonatal Thyroid Stimulating Hormone
WHO: World Health Organisation
CH: Congenital hypothyroidism
TBG: Thyroid-binding globulin
