# Supplementary material for: Neonatal heel prick screening TSH concentration in the Netherlands as indicator of iodine status
Source: Nutr J. 2021 Jul 4;20:63. doi: 10.1186/s12937-021-00722-4 (PMC8256570; doi:10.1186/s12937-021-00722-4)
Supplement: Supplementary file 1 — Additional file 1. [file 12937_2021_722_MOESM1_ESM.docx]

**Online Supplemental Material Table 1** General characteristics of the study population by calendar year presented as median (P5-P95) or proportion (%). p-value of Chi-square test or Spearman correlation.

|  |  | **whole population** | | | | | | | | |  |
| --- | --- | --- | --- | --- | --- | --- | --- | --- | --- | --- | --- |
|  |  | **2007** | **2008** | **2009** | **2010** | **2011** | **2012** | **2013** | **2014** | **2015** | **p-value** |
| **N** |  | 165368 | 168950 | 141632 | 157463 | 165612 | 161490 | 157036 | 160936 | 157018 |  |
| **gender** | **male** | 51 | 51 | 51 | 51 | 52 | 52 | 52 | 51 | 51 | χ^2^ = 11, 8 d.f., p=0.2186 |
| **birth season** | **spring** | 24 | 24 | 26 | 21 | 24 | 24 | 24 | 24 | 24 | χ^2^ = 1765, 24 d.f., p<0.0001 |
|  | **summer** | 24 | 25 | 24 | 24 | 25 | 25 | 25 | 25 | 25 |  |
|  | **autumn** | 27 | 27 | 26 | 28 | 26 | 26 | 27 | 27 | 27 |  |
|  | **winter** | 25 | 24 | 24 | 27 | 24 | 24 | 24 | 25 | 25 |  |
| **age heel prick** | **day 3** | 4 | 6 | 7 | 8 | 8 | 9 | 9 | 9 | 10 | χ^2^ = 8397, 32 d.f., p<0.0001 |
|  | **day 4** | 50 | 52 | 49 | 50 | 51 | 51 | 51 | 50 | 48 |  |
|  | **day 5** | 25 | 24 | 25 | 24 | 23 | 24 | 24 | 24 | 24 |  |
|  | **day 6** | 16 | 15 | 16 | 15 | 15 | 15 | 14 | 15 | 15 |  |
|  | **day 7** | 4 | 4 | 3 | 3 | 3 | 3 | 2 | 2 | 3 |  |
| **laboratory** | **Amsterdam** | 20 | 20 | 23 | 22 | 20 | 20 | 20 | 20 | 20 | χ^2^ = 26176, 32 d.f., p<0.0001 |
|  | **Bilthoven** | 20 | 20 | 24 | 22 | 20 | 20 | 20 | 20 | 20 |  |
|  | **Capelle** | 24 | 24 | 29 | 26 | 25 | 25 | 25 | 25 | 25 |  |
|  | **Tilburg** | 19 | 19 | 3 | 13 | 19 | 19 | 19 | 19 | 19 |  |
|  | **Zwolle** | 17 | 17 | 20 | 18 | 17 | 17 | 16 | 16 | 16 |  |
| **pregnancy duration (days)** |  | 280 (263-293) | 280 (263-293) | 280 (262-292) | 280 (262-292) | 280 (262-291) | 279 (262-291) | 279 (262-291) | 279 (262-291) | 279 (262-291) | ρ=-0.03 p<0.0001 |
| **birth weight (kg)** |  | 3.5 (2.8-4.4) | 3.5 (2.8-4.4) | 3.5 (2.8-4.4) | 3.5 (2.8-4.3) | 3.5 (2.8-4.3) | 3.5 (2.8-4.3) | 3.5 (2.8-4.3) | 3.5 (2.8-4.3) | 3.5 (2.8-4.3) | ρ=-0.05 p<0.0001 |
|  |  |  |  |  |  |  |  |  |  |  |  |
|  |  | **TSH subpopulation** | | | | | | | | |  |
|  |  | **2007** | **2008** | **2009** | **2010** | **2011** | **2012** | **2013** | **2014** | **2015** |  |
| **N** |  | 31249 | 31087 | 26208 | 28438 | 30629 | 29101 | 28052 | 30131 | 29561 |  |
| **gender** | **male** | 58 | 59 | 59 | 59 | 60 | 60 | 60 | 59 | 60 | χ^2^ = 27, 8 d.f., p=0.0006 |
| **birth season** | **spring** | 24 | 24 | 26 | 20 | 24 | 25 | 24 | 24 | 24 | χ^2^ = 564, 24 d.f., p<0.0001 |
|  | **summer** | 24 | 24 | 24 | 23 | 25 | 25 | 24 | 24 | 25 |  |
|  | **autumn** | 27 | 27 | 26 | 30 | 29 | 26 | 28 | 27 | 27 |  |
|  | **winter** | 25 | 24 | 24 | 28 | 23 | 24 | 24 | 25 | 24 |  |
| **age heel prick** | **day 3** | 4 | 5 | 7 | 7 | 7 | 8 | 8 | 8 | 9 | χ^2^ = 1743, 32 d.f., p<0.0001 |
|  | **day 4** | 46 | 47 | 44 | 46 | 47 | 47 | 48 | 47 | 44 |  |
|  | **day 5** | 24 | 24 | 25 | 24 | 23 | 23 | 24 | 23 | 24 |  |
|  | **day 6** | 19 | 19 | 19 | 19 | 18 | 18 | 17 | 18 | 19 |  |
|  | **day 7** | 6 | 6 | 5 | 4 | 4 | 4 | 3 | 3 | 4 |  |
| **laboratory** | **Amsterdam** | 20 | 20 | 24 | 22 | 19 | 19 | 18 | 18 | 19 | χ^2^ = 5066, 32 d.f., p<0.0001 |
|  | **Bilthoven** | 21 | 20 | 25 | 22 | 21 | 22 | 20 | 20 | 21 |  |
|  | **Capelle** | 23 | 23 | 29 | 25 | 24 | 24 | 26 | 27 | 26 |  |
|  | **Tilburg** | 19 | 19 | 3 | 13 | 19 | 20 | 19 | 18 | 18 |  |
|  | **Zwolle** | 17 | 17 | 20 | 18 | 17 | 17 | 17 | 16 | 16 |  |
| **pregnancy duration (days)** |  | 279 (261-292) | 278 (260-292) | 278 (260-292) | 277 (260-291) | 278 (260-291) | 277 (260-290) | 277 (259-290) | 277 (260-290) | 277 (260-290) | ρ=-0.03 p<0.0001 |
| **birth weight (kg)** |  | 3.4 (2.7-4.3) | 3.4 (2.7-4.3) | 3.4 (2.7-4.3) | 3.4 (2.7-4.2) | 3.4 (2.7-4.2) | 3.4 (2.7-4.2) | 3.4 (2.7-4.2) | 3.4 (2.7-4.2) | 3.4 (2.7-4.2) | ρ=-0.06 p<0.0001 |

**Online Supplemental Material Table 2** T4 concentration (nmol/L) median (P5-P95), p-value of Kruskal-Wallis H-test

|  |  | **whole population** | | | | | | | | | | | | | | | | |  |
| --- | --- | --- | --- | --- | --- | --- | --- | --- | --- | --- | --- | --- | --- | --- | --- | --- | --- | --- | --- |
|  |  | **2007** | | **2008** | | **2009** | | **2010** | | **2011** | | **2012** | | **2013** | **2014** | | **2015** | | **p-value** |
| **gender** | **male** | 85 (56-124) | | 81 (55-116) | | 84 (57-119) | | 89 (61-125) | | 86 (59-123) | | 86 (59-121) | | 85 (58-120) | 85 (57-120) | | 89 (60-126) | | χ^2^ = 24, 8 d.f., p=0.0026 |
|  | **female** | 88 (60-128) | | 84 (59-119) | | 87 (60-122) | | 92 (65-128) | | 89 (63-127) | | 89 (62-125) | | 88 (62-124) | 88 (61-124) | | 92 (65-130) | | χ^2^ = 47, 8 d.f., p<0.0001 |
|  |  | χ^2^ = 981-1445^*^, 1 d.f., p<0.0001 | | | | | | | | | | | | | | | | |  |
|  |  |  | |  | |  | |  | |  | |  | |  |  | |  | |  |
| **birth season** | **spring** | 91 (61-130) | | 86 (59-124) | | 90 (63-126) | | 91 (64-126) | | 90 (64-128) | | 91 (69-128) | | 91 (63-127) | 88 (61-125) | | 92 (63-132) | | χ^2^ = 10, 8 d.f., p=0.2656 |
|  | **summer** | 86 (58-123) | | 80 (55-113) | | 85 (58-120) | | 92 (64-128) | | 86 (60-123) | | 85 (58-122) | | 86 (61-121) | 87 (60-122) | | 91 (63-130) | | χ^2^ = 12, 8 d.f., p=0.1339 |
|  | **autumn** | 84 (57-123) | | 79 (54-112) | | 80 (54-112) | | 88 (61-123) | | 86 (60-124) | | 84 (59-118) | | 83 (58-118) | 83 (57-118) | | 88 (60-123) | | χ^2^ = 24, 8 d.f., p=0.0027 |
|  | **winter** | 87 (58-127) | | 86 (60-120) | | 87 (61-121) | | 91 (64-129) | | 88 (62-124) | | 88 (62-124) | | 85 (60-121) | 88 (60-124) | | 92 (64-129) | | χ^2^ = 21, 8 d.f., p=0.0072 |
|  |  | χ^2^ = 1, 3 d.f., p=0.7394 | χ^2^ = 1, 3 d.f., p=0.8047 | | χ^2^ = 11, 3 d.f., p=0.0105 | | χ^2^ = 3, 3 d.f., p=0.4475 | | χ^2^ = 17, 3 d.f., p=0.0006 | | χ^2^ = 6, 3 d.f., p=0.1099 | | χ^2^ = 4, 3 d.f., p0.2657 | | | χ^2^ = 6, 3 d.f., p=0.1118 | | χ^2^ = 3, 3 d.f., p=0.3956 |  |
|  |  |  | |  | |  | |  | |  | |  | |  |  | |  | |  |
| **age heel prick** | **day 3** | 89 (60-131) | | 85 (59-122) | | 87 (60-123) | | 92 (63-130) | | 90 (63-129) | | 89 (61-127) | | 89 (62-126) | 90 (61-128) | | 93 (63-134) | | χ^2^ = 35, 8 d.f., p<0.0001 |
|  | **day 4** | 88 (59-128) | | 84 (58-120) | | 87 (60-123) | | 92 (64-129) | | 89 (62-127) | | 89 (61-126) | | 88 (61-124) | 88 (60-124) | | 92 (63-131) | | χ^2^ = 58, 8 d.f., p<0.0001 |
|  | **day 5** | 87 (59-125) | | 82 (56-116) | | 85 (58-119) | | 90 (63-126) | | 87 (61-123) | | 87 (60-121) | | 86 (60-120) | 86 (60-121) | | 90 (62-126) | | χ^2^ = 22, 8 d.f., p=0.0047 |
|  | **day 6** | 83 (56-120) | | 79 (55-111) | | 81 (56-115) | | 87 (61-121) | | 84 (59-118) | | 83 (58-117) | | 83 (58-115) | 82 (57-114) | | 86 (60-120) | | χ^2^ = 37, 8 d.f., p<0.0001 |
|  | **day 7** | 79 (54-115) | | 75 (52-105) | | 78 (54-110) | | 84 (56-117) | | 69 (56-109) | | 77 (55-108) | | 79 (56-110) | 79 (55-111) | | 83 (58-115) | | χ^2^ = 15, 8 d.f., p=0.0595 |
|  |  | χ^2^ = 1436-2490^*^, 4 d.f., p<0.0001 | | | | | | | | | | | | | | | | |  |
|  |  |  | |  | |  | |  | |  | |  | |  |  | |  | |  |
| **laboratory** | **Amsterdam** | 81 (55-116) | | 79 (54-112) | | 82 (56-114) | | 87 (61-121) | | 82 (58-116) | | 80 (57-113) | | 82 (58-114) | 81 (57-113) | | 86 (61-119) | | χ^2^ = 6, 8 d.f., p=06034 |
|  | **Bilthoven** | 92 (60-134) | | 86 (59-124) | | 87 (57-125) | | 94 (65-132) | | 90 (61-129) | | 88 (60-123) | | 89 (61-125) | 90 (61-126) | | 94 (63-131) | | χ^2^ = 23, 8 d.f., p=0.0030 |
|  | **Capelle** | 87 (59-126) | | 83 (57-117) | | 87 (60-120) | | 90 (63-125) | | 88 (61-122) | | 87 (60-123) | | 87 (61-122) | 88 (59-127) | | 90 (60-129) | | χ^2^ = 17, 8 d.f., p=0.0355 |
|  | **Tilburg** | 84 (57-120) | | 81 (56-114) | | 90 (63-128) | | 88 (62-123) | | 90 (63-127) | | 91 (62-127) | | 85 (59-120) | 86 (60-118) | | 91 (63-125) | | χ^2^ = 36, 8 d.f., p<0.0001 |
|  | **Zwolle** | 91 (61-130) | | 85 (59-121) | | 86 (60-121) | | 91 (64-129) | | 90 (63-130) | | 91 (64-128) | | 89 (62-127) | 87 (60-123) | | 94 (65-136) | | χ^2^ = 42, 8 d.f., p<0.0001 |
|  |  | χ^2^ = 22, 4 d.f., p=0.0002 | | χ^2^ = 20, 4 d.f., p=0.0005 | | χ^2^ = 16, 4 d.f., p=0.0027 | | χ^2^ = 13, 4 d.f., p=0.0131 | | χ^2^ = 11, 4 d.f., p=0.0219 | | χ^2^ = 17, 4 d.f., p=0.0024 | | χ^2^ = 7, 4 d.f., p=0.1375 | χ^2^ = 25, 4 d.f., p<0.0001 | | χ^2^ = 32, 4 d.f., p<0.0001 | |  |
|  |  |  | |  | |  | |  | |  | |  | |  |  | |  | |  |
| **pregnancy duration** | **253-275 days** | 84 (56-123) | | 80 (55-115) | | 83 (56-118) | | 88 (61-124) | | 85 (59-122) | | 84 (58-120) | | 83 (58-119) | 83 (57-119) | | 88 (60-125) | | χ^2^ = 36, 8 d.f., p<0.0001 |
|  | **276-283 days** | 87 (59-126) | | 83 (57-118) | | 86 (59-121) | | 91 (64-127) | | 88 (62-125) | | 88 (61-123) | | 87 (61-123) | 87 (60-123) | | 91 (63-129) | | χ^2^ = 97, 8 d.f., p<0.0001 |
|  | **284-313 days** | 89 (60-128) | | 84 (58-120) | | 87 (60-123) | | 93 (65-129) | | 90 (63-128) | | 89 (62-126) | | 89 (62-125) | 89 (62-125) | | 94 (65-132) | | χ^2^ = 123, 8 d.f., p<0.0001 |
|  |  | χ^2^ = 1498-2657^*^, 2 d.f., p<0.0001 | | | | | | | | | | | | | | | | |  |
|  |  |  | |  | |  | |  | |  | |  | |  |  | |  | |  |
| **birth weight** | **2.5-3.3 kg** | 84 (56-123) | | 80 (55-115) | | 83 (57-118) | | 88 (61-124) | | 85 (59-122) | | 85 (58-121) | | 84 (58-119) | 84 (57-119) | | 88 (60-125) | | χ^2^ = 12, 8 d.f., p=0.1576 |
|  | **3.3-3.7 kg** | 86 (58-125) | | 82 (57-117) | | 85 (59-120) | | 90 (63-126) | | 88 (62-125) | | 87 (60-123) | | 87 (61-122) | 87 (60-122) | | 91 (63-128) | | χ^2^ = 65, 8 d.f., p<0.0001 |
|  | **3.7-7.7 kg** | 89 (61-129) | | 85 (59-121) | | 88 (60-123) | | 93 (65-130) | | 90 (63-128) | | 89 (62-126) | | 89 (62-125) | 89 (62-126) | | 93 (65-132) | | χ^2^ = 57, 8 d.f., p<0.0001 |
|  |  | χ^2^ = 1606-2211^*^, 2 d.f., p<0.0001 | | | | | | | | | | | | | | | | |  |
|  |  |  | |  | |  | |  | |  | |  | |  |  | |  | |  |
|  |  | **TSH subpopulation** | | | | | | | | | | | | | | | | |  |
|  |  | **2007** | | **2008** | | **2009** | | **2010** | | **2011** | | **2012** | | **2013** | **2014** | | **2015** | |  |
| **gender** | **male** | 63 (47-77) | | 61 (42-74) | | 64 (46-77) | | 68 (51-79) | | 66 (49-77) | | 65 (48-77) | | 65 (48-76) | 64 (47-76) | | 67 (49-80) | | χ^2^ = 111, 8 d.f., p<0.0001 |
|  | **female** | 64 (49-77) | | 63 (48-74) | | 65 (49-77) | | 68 (53-80) | | 67 (52-78) | | 66 (51-77) | | 65 (51-77) | 65 (50-76) | | 68 (53-80) | | χ^2^ = 45, 8 d.f., p<0.0001 |
|  |  | χ^2^ = 143-233*, 1 d.f., p<0.0001 | | | | | | | | | | | | | | | | |  |
|  |  |  | |  | |  | |  | |  | |  | |  |  | |  | |  |
| **birth season** | **spring** | 67 (50-80) | | 64 (48-76) | | 68 (52-80) | | 68 (52-79) | | 69 (52-80) | | 69 (52-79) | | 68 (52-79) | 66 (50-78) | | 69 (51-82) | | χ^2^ = 49, 8 d.f., p<0.0001 |
|  | **summer** | 63 (47-75) | | 60 (45-71) | | 64 (47-75) | | 69 (52-80) | | 65 (49-76) | | 64 (48-77) | | 65 (50-76) | 65 (49-75) | | 68 (51-80) | | χ^2^ = 50, 8 d.f., p<0.0001 |
|  | **autumn** | 62 (47-76) | | 60 (45-71) | | 60 (44-70) | | 66 (50-76) | | 65 (49-76) | | 63 (48-73) | | 63 (48-74) | 62 (47-73) | | 65 (49-76) | | χ^2^ = 48, 8 d.f., p<0.0001 |
|  | **winter** | 63 (47-77) | | 65 (49-76) | | 66 (49-76) | | 69 (53-81) | | 67 (50-78) | | 67 (50-77) | | 64 (49-76) | 66 (50-77) | | 69 (52-81) | | χ^2^ = 47, 8 d.f., p<0.0001 |
|  |  | χ^2^ = 3, 3 d.f., p=0.3522 | | χ^2^ = 20, 3 d.f., p=0.0001 | | χ^2^ = 3, 3 d.f., p=0.4616 | | χ^2^ = 1, 3 d.f., p=0.7245 | | χ^2^ = 7, 3 d.f., p=0.0803 | | χ^2^ = 3, 3 d.f., p=0.3662 | | χ^2^ = 6, 3 d.f., p=0.1363 | χ^2^ = 12, 3 d.f., p=0.0.0064 | | χ^2^ = 2, 3 d.f., p=0.6569 | |  |
|  |  |  | |  | |  | |  | |  | |  | |  |  | |  | |  |
| **age heel prick** | **day 3** | 65 (48-78) | | 63 (47-75) | | 65 (49-76) | | 68 (51-79) | | 67 (50-78) | | 66 (49-77) | | 66 (49-77) | 65 (48-77) | | 68 (50-81) | | χ^2^ = 32, 8 d.f., p<0.0001 |
|  | **day 4** | 64 (48-77) | | 62 (46-74) | | 65 (48-77) | | 68 (52-79) | | 67 (50-78) | | 66 (50-78) | | 65 (49-77) | 65 (48-76) | | 68 (50-81) | | χ^2^ = 76, 8 d.f., p<0.0001 |
|  | **day 5** | 64 (48-77) | | 62 (46-74) | | 64 (47-76) | | 68 (51-79) | | 66 (50-77) | | 65 (49-77) | | 65 (49-76) | 65 (49-76) | | 67 (51-80) | | χ^2^ = 34, 8 d.f., p<0.0001 |
|  | **day 6** | 63 (47-77) | | 61 (47-73) | | 63 (46-76) | | 67 (52-78) | | 65 (50-78) | | 65 (49-76) | | 64 (49-76) | 64 (48-75) | | 67 (51-79) | | χ^2^ = 29, 8 d.f., p=0.0003 |
|  | **day 7** | 62 (47-76) | | 60 (45-72) | | 63 (46-76) | | 66 (52-79) | | 64 (49-76) | | 62 (48-75) | | 63 (47-76) | 63 (48-74) | | 67 (50-80) | | χ^2^ = 17, 8 d.f., p=0.0299 |
|  |  | χ^2^ = 12, 4 d.f., p=0.0160 | | χ^2^ = 14, 4 d.f., p=0.0073 | | χ^2^ = 10, 4 d.f., p=0.0396 | | χ^2^ = 9, 4 d.f., p=0.0515 | | χ^2^ = 10, 4 d.f., p=0.0481 | | χ^2^ = 7, 4 d.f., p=0.1249 | | χ^2^ = 15, 4 d.f., p=0.0038 | χ^2^ = 3, 4 d.f., p=0.5242 | | χ^2^ = 19, 4 d.f., p=0.0009 | |  |
|  |  |  | |  | |  | |  | |  | |  | |  |  | |  | |  |
| **laboratory** | **Amsterdam** | 60 (45-71) | | 59 (45-70) | | 61 (45-73) | | 66 (50-75) | | 62 (48-72) | | 61 (47-71) | | 62 (47-73) | 61 (47-72) | | 65 (50-76) | | χ^2^ = 57, 8 d.f., p<0.0001 |
|  | **Bilthoven** | 67 (49-80) | | 64 (47-76) | | 65 (45-80) | | 71 (52-82) | | 67 (50-81) | | 65 (49-77) | | 67 (50-79) | 67 (49-78) | | 69 (51-81) | | χ^2^ = 78, 8 d.f., p<0.0001 |
|  | **Capelle** | 64 (48-76) | | 62 (47-74) | | 65 (49-75) | | 68 (52-78) | | 66 (50-76) | | 65 (49-77) | | 65 (49-75) | 65 (48-77) | | 67 (49-79) | | χ^2^ = 208, 8 d.f., p<0.0001 |
|  | **Tilburg** | 62 (47-74) | | 61 (46-72) | | 69 (53-78) | | 67 (52-77) | | 68 (52-79) | | 68 (51-79) | | 64 (49-77) | 64 (49-74) | | 68 (52-79) | | χ^2^ = 56, 8 d.f., p<0.0001 |
|  | **Zwolle** | 67 (51-80) | | 64 (48-75) | | 65 (50-77) | | 69 (53-80) | | 69 (52-79) | | 69 (52-79) | | 67 (52-77) | 65 (49-77) | | 71 (54-84) | | χ^2^ = 5, 8 d.f., p=0.7582 |
|  |  | χ^2^ = 7, 4 d.f., p=0.1373 | | χ^2^ = 3, 4 d.f., p=0.6343 | | χ^2^ = 13, 4 d.f., p=0.0101 | | χ^2^ = 17, 4 d.f., p=0.0016 | | χ^2^ = 19, 4 d.f., p=0.0006 | | χ^2^ = 66, 4 d.f., p<0.0001 | | χ^2^ = 24, 4 d.f., p<0.0001 | χ^2^ = 121, 4 d.f., p<0.0001 | | χ^2^ = 115, 4 d.f., p<0.0001 | |  |
|  |  |  | |  | |  | |  | |  | |  | |  |  | |  | |  |
| **pregnancy duration** | **253-275 days** | 63 (46-77) | | 62 (45-74) | | 64 (47-76) | | 67 (51-79) | | 66 (49-78) | | 65 (48-77) | | 65 (48-76) | 64 (48-76) | | 67 (50-80) | | χ^2^ = 58, 8 d.f., p<0.0001 |
|  | **276-283 days** | 64 (48-77) | | 62 (47-74) | | 64 (48-77) | | 68 (53-79) | | 67 (51-78) | | 66 (50-77) | | 65 (50-77) | 65 (49-76) | | 68 (52-80) | | χ^2^ = 42, 8 d.f., p<0.0001 |
|  | **284-313 days** | 65 (49-77) | | 62 (47-74) | | 65 (48-77) | | 68 (52-79) | | 67 (51-78) | | 66 (50-77) | | 65 (50-77) | 65 (49-76) | | 68 (52-81) | | χ^2^ = 48, 8 d.f., p<0.0001 |
|  |  | χ^2^ = 70-171*, 2 d.f., p<0.0001 | | | | | | | | | | | | | | | | |  |
|  |  |  | |  | |  | |  | |  | |  | |  |  | |  | |  |
| **birth weight** | **2.5-3.3 kg** | 63 (47-77) | | 62 (46-74) | | 64 (47-76) | | 67 (51-79) | | 66 (49-77) | | 65 (48-77) | | 64 (48-77) | 64 (47-76) | | 67 (50-80) | | χ^2^ = 55, 8 d.f., p<0.0001 |
|  | **3.3-3.7 kg** | 64 (48-77) | | 62 (47-74) | | 64 (47-77) | | 68 (52-79) | | 66 (51-78) | | 66 (50-77) | | 65 (50-76) | 65 (49-76) | | 68 (51-80) | | χ^2^ = 57, 8 d.f., p<0.0001 |
|  | **3.7-7.7 kg** | 64 (48-77) | | 62 (47-74) | | 64 (48-77) | | 68 (52-80) | | 67 (51-78) | | 66 (50-77) | | 65 (50-77) | 65 (49-76) | | 68 (52-81) | | χ^2^ = 50, 8 d.f., p<0.0001 |
|  |  | χ^2^ = 45-110*, 2 d.f., p<0.0001 | | | | | | | | | | | | | | | | |  |

* Range of χ^2^ value between years

**Online Supplemental Material Table 3a**. Proportion (%) new-borns with a specific rounded TSH value in heel prick blood by calendar year

| TSH mUI/L | **2007** | **2008** | **2009** | **2010** | **2011** | **2012** | **2013** | **2014** | **2015** |
| --- | --- | --- | --- | --- | --- | --- | --- | --- | --- |
| 1 | 72.19 | 73.74 | 73.92 | 68.9 | 74.15 | 71.74 | 62.39 | 50.73 | 50.46 |
| 2 | 19.6 | 17.95 | 17.31 | 20.53 | 17.66 | 17 | 29.92 | 41.71 | 38.53 |
| 3 | 5.46 | 5.14 | 5.29 | 6.13 | 4.93 | 7.64 | 4.79 | 4.47 | 6.32 |
| 4 | 1.53 | 1.72 | 1.87 | 2.2 | 1.67 | 2.05 | 1.44 | 1.57 | 2.55 |
| 5 | 0.59 | 0.65 | 0.71 | 0.96 | 0.71 | 0.64 | 0.67 | 0.72 | 1.07 |
| 6 | 0.23 | 0.24 | 0.34 | 0.49 | 0.29 | 0.32 | 0.34 | 0.37 | 0.51 |
| 7 | 0.1 | 0.13 | 0.15 | 0.18 | 0.14 | 0.13 | 0.11 | 0.09 | 0.12 |
| 8 | 0.07 | 0.09 | 0.06 | 0.14 | 0.08 | 0.11 | 0.07 | 0.05 | 0.09 |
| 9 | 0.06 | 0.07 | 0.06 | 0.09 | 0.08 | 0.04 | 0.03 | 0.04 | 0.06 |
| 10 | 0.01 | 0.02 | 0.04 | 0.04 | 0.06 | 0.04 | 0.01 | 0.04 | 0.05 |
| >10 | 0.15 | 0.24 | 0.24 | 0.32 | 0.23 | 0.29 | 0.22 | 0.22 | 0.25 |
| >5 | 0.62 | 0.79 | 0.89 | 1.26 | 0.88 | 0.93 | 0.78 | 0.81 | 1.08 |

| **Online Supplemental Material Table 3b**. Proportion (%) new-borns with a specific rounded TSH value in heel prick blood by age of heel prick sampling | | | | | |
| --- | --- | --- | --- | --- | --- |
| TSH mUI/L | **Amsterdam** | **Bilthoven** | **Capelle** | **Tilburg** | **Zwolle** |
| TSH 1 mUI/L | 54.86 | 68.5 | 65.68 | 66.71 | 78.17 |
| TSH 2 mIU/L | 26.72 | 26.11 | 27.61 | 24.22 | 15.72 |
| TSH 3 mIU/L | 11.04 | 3.08 | 4.18 | 6.2 | 3.73 |
| 4 | 4.18 | 1.11 | 1.23 | 1.55 | 1.2 |
| 5 | 1.59 | 0.46 | 0.54 | 0.61 | 0.55 |
| 6 | 0.72 | 0.26 | 0.29 | 0.25 | 0.2 |
| 7 | 0.24 | 0.09 | 0.11 | 0.08 | 0.12 |
| 8 | 0.16 | 0.05 | 0.07 | 0.1 | 0.05 |
| 9 | 0.1 | 0.07 | 0.03 | 0.04 | 0.06 |
| 10 | 0.05 | 0.05 | 0.03 | 0.03 | 0.01 |
| >10 | 0.34 | 0.21 | 0.23 | 0.21 | 0.2 |
| >5 | 1.61 | 0.73 | 0.76 | 0.71 | 0.64 |

| **Online Supplemental Material Table 3c**. Proportion (%) new-borns with a specific rounded TSH value in heel prick blood by age of heel prick sampling | | | | | |
| --- | --- | --- | --- | --- | --- |
| TSH mUI/L | **day 3** | **day 4** | **day 5** | **day 6** | **day 7** |
| 1 | 62.86 | 66.14 | 68.42 | 67.46 | 60.56 |
| 2 | 26.92 | 24.58 | 23.89 | 23.56 | 27.02 |
| 3 | 6.06 | 5.6 | 4.8 | 5.69 | 8.16 |
| 4 | 2.13 | 1.88 | 1.54 | 1.86 | 2.56 |
| 5 | 0.99 | 0.77 | 0.63 | 0.68 | 0.96 |
| 6 | 0.43 | 0.4 | 0.25 | 0.31 | 0.33 |
| 7 | 0.12 | 0.14 | 0.12 | 0.1 | 0.14 |
| 8 | 0.13 | 0.09 | 0.07 | 0.07 | 0.09 |
| 9 | 0.1 | 0.07 | 0.04 | 0.04 | 0.04 |
| 10 | 0.03 | 0.04 | 0.03 | 0.02 | 0.01 |
| >10 | 0.22 | 0.28 | 0.22 | 0.2 | 0.14 |
| >5 | 1.03 | 1.02 | 0.73 | 0.74 | 0.75 |

**Online Supplemental Material Figure 1.** Odds ratio and their 95% confidence intervals of exceeding blood spot screening cut off (nTSH > 5mIU/L versus nTSH<=5mIU/L) for year separate for laboratory for age 7 days. Multivariate analyses with the following characteristics (reference value): year (2007), laboratory (Zwolle), age of heel prick blood sampling (4 days), gender (male), birth weight category (middle: 3.31-3.71 kg), pregnancy duration category (middle: 276-284 days), season (spring), and interaction terms: year*laboratory, age*year, age*laboratory
